# Magnesium Sulfate Protects Against the Bioenergetic Consequences of Chronic Glutamate Receptor Stimulation

**DOI:** 10.1371/journal.pone.0079982

**Published:** 2013-11-13

**Authors:** Pascaline Clerc, Christina A. Young, Evan A. Bordt, Alina M. Grigore, Gary Fiskum, Brian M. Polster

**Affiliations:** Department of Anesthesiology and Center for Shock, Trauma and Anesthesiology Research (STAR), University of Maryland School of Medicine, Baltimore, Maryland, United States of America; University of S. Florida College of Medicine, United States of America

## Abstract

Extracellular glutamate is elevated following brain ischemia or trauma and contributes to neuronal injury. We tested the hypothesis that magnesium sulfate (MgSO_4_, 3 mM) protects against metabolic failure caused by excitotoxic glutamate exposure. Rat cortical neuron preparations treated in medium already containing a physiological concentration of Mg^2+^ (1 mM) could be segregated based on their response to glutamate (100 µM). Type I preparations responded with a decrease or small transient increase in oxygen consumption rate (OCR). Type II neurons responded with >50% stimulation in OCR, indicating a robust response to increased energy demand without immediate toxicity. Pre-treatment with MgSO_4_ improved the initial bioenergetic response to glutamate and ameliorated subsequent loss of spare respiratory capacity, measured following addition of the uncoupler FCCP, in Type I but not Type II neurons. Spare respiratory capacity in Type I neurons was also improved by incubation with MgSO_4_ or NMDA receptor antagonist MK801 in the absence of glutamate treatment. This finding indicates that the major difference between Type I and Type II preparations is the amount of endogenous glutamate receptor activity. Incubation of Type II neurons with 5 µM glutamate prior to excitotoxic (100 µM) glutamate exposure recapitulated a Type I phenotype. MgSO_4_ protected against an excitotoxic glutamate-induced drop in neuronal ATP both with and without prior 5 µM glutamate exposure. Results indicate that MgSO_4_ protects against chronic moderate glutamate receptor stimulation and preserves cellular ATP following treatment with excitotoxic glutamate.

## Introduction

Magnesium (Mg^2+^) is present both intracellularly and extracellularly in the nervous system. It plays an essential role as a messenger and modulator of enzymatic activity. Mg^2+^ is essential for the activity of over 300 enzymes, including α-ketoglutarate dehydrogenase and ATP synthase within mitochondria [1], [2]. In the setting of traumatic brain injury, Mg^2+^ therapy protects against mitochondrial respiratory dysfunction and improves cytosolic phosphorylation potential [3], [4].

Numerous animal models have demonstrated the neuroprotective properties of Mg^2+^ administered prophylactically or immediately after cerebral ischemia. In a rat model of diffuse brain injury, intravenously or intramuscularly administered Mg^2+^ penetrated the blood brain barrier, increased brain intracellular free Mg^2+^, and improved the overall neuronal energetic performance [4]. In a rat model of transient global ischemia, intravenous MgSO_4_ effectively ameliorated CA1 hippocampal cell death when combined with moderate (35°C) hypothermia [5]. Moreover, in patients presenting with stroke, cerebrospinal fluid Mg^2+^ levels are predictive of both neurological outcome and neurological improvement [6]. The mechanisms by which magnesium exerts its beneficial effect still need to be elucidated.

Glutamate is released in the brain during and following an ischemic or traumatic insult [7]. Glutamate stimulates O_2_ consumption by cultured rat cortical or cerebellar granule neurons, primarily due to increased energy demand [8]–[10]. Sodium flux through AMPA- and NMDA-type glutamate receptors accelerates ATP hydrolysis by the Na^+^/K^+^ ATPase, constituting much of the increased demand for ATP [11]. NMDA receptor-mediated calcium entry is pivotal for excitotoxic cell death [12], [13]. However, decreasing mitochondrial spare respiratory capacity, which is the difference between basal and maximal respiration, also potentiates glutamate excitotoxicity [9] whereas delivery of exogenous energy substrates delays mitochondrial compromise [14]. These findings indicate that glutamate excitotoxicity has a metabolic component. Extracellular Mg^2+^ exerts a voltage-dependent block of NMDA receptors [15] and NMDA receptor inhibition is frequently cited as an explanation for the neuroprotective effect of Mg^2+^ [16]. However, excitotoxic glutamate levels are predicted to remove the Mg^2+^ block of NMDA receptors via AMPA receptor-mediated depolarization.

In this study we tested the hypothesis that MgSO_4_ pre-treatment protects against mitochondrial bioenergetic failure caused by excitotoxic glutamate exposure through NMDA receptor-independent mechanism(s). Bioenergetic function was evaluated by two key parameters: 1) the initial change in O_2_ consumption rate (OCR) in response to glutamate and 2) the change in respiratory capacity following transient glutamate receptor stimulation. Respiratory capacity was defined as the maximum respiration measured in the presence of the uncoupler FCCP and excess exogenous substrate (10 mM pyruvate). Relative respiratory capacity was defined as maximum respiration normalized to the basal O_2_ consumption rate. Results suggest that MgSO_4_ pre-treatment protects against bioenergetic changes due to chronic moderate glutamate receptor stimulation but not due to acute excitotoxic glutamate receptor stimulation, primarily by NMDA receptor-dependent mechanisms. However, MgSO_4_ preserved neuronal ATP levels even though it was unable to rescue the reduction in relative respiratory capacity caused by an excitotoxic concentration of glutamate.

## Materials and Methods

### Materials

Cell culture supplies were purchased from Invitrogen (Grand Island, NY). All other reagents were obtained from Sigma-Aldrich (St. Louis, MO). Pyruvate was made fresh from powder and pH-adjusted for each individual experiment. Other reagents were diluted from concentrated pH-adjusted stocks stored at −20°C.

### Preparation of primary neurons

Ethics Statement: All procedures were approved by the University of Maryland Institutional Animal Care and Use Committee (IACUC protocol # 1109008) and were in accordance with the NIH Guide for the Care and Use of Laboratory Animals. Primary E18 rat cortical neurons were prepared by trypsin dissociation [17], [18] and plated and maintained in V7 microplates (Seahorse Bioscience) at a density of 0.8×10^5^ to 1×10^5^ cells/well (0.32 cm^2^) as described [19]. Cytosine arabinofuranoside (5 µM) was added at 4 days *in vitro* (DIV) to inhibit glial proliferation [20] and 2∶7 fresh Neurobasal/B27 culture medium was added on DIV 6. Neurons were maintained at 37°C in a humidified atmosphere of 95% air/5% CO_2_ and were used for experiments at 11–15 DIV. Astrocytic contamination of neuronal cultures was less than 5% as determined by GFAP immunocytochemistry to identify astrocytes and NeuN counterstaining to identify neurons.

### Measurement of O_2_ consumption by XF24 microplate-based respirometry

O_2_ consumption measurements were made using an XF24 Extracellular Flux Analyzer (Seahorse Bioscience) as previously described [19]. Artificial cerebrospinal fluid (aCSF) assay medium consisted of 120 mM NaCl, 3.5 mM KCl, 1.3 mM CaCl_2_, 0.4 mM KH_2_PO_4_, 1 mM MgCl_2_, 15 mM glucose, 4 mg/ml fatty acid free bovine serum album, and 5 HEPES, pH 7.2. For low Ca^2+^ aCSF, 1.3 mM CaCl_2_ was replaced by 1.86 mM CaCl_2_ and 5 mM EGTA to yield ∼100 nM free Ca^2+^ [14]. Neurons were incubated with or without treatment (e.g. MgSO_4_) in a CO_2_-free 37°C incubator for one hour prior to measurements. Treatments were maintained throughout the experiments and were as indicated in the figure legends. XF assays consisted of cycles of 3 min mix, 2 min wait, 2 min measurement and were performed as described [19], [21] at 37°C.

The protonophore carbonyl cyanide 4-(trifluoromethoxy) phenylhydrazone (FCCP, 3 µM) was added to measure uncoupled respiration and the complex III inhibitor antimycin A (1 µM) was used to inhibit O_2_ consumption by the mitochondrial electron transport chain.

### ATP quantification

Neuronal ATP levels were analyzed using the ATP bioluminescent somatic cell assay kit for cellular ATP determination (Sigma-Aldrich). Neurons were incubated with or without treatment (e.g. 3 mM MgSO_4_±5 µM glutamate) in a CO_2_-free 37°C incubator for one hour. Glutamate (100 µM, plus 10 µM glycine) or vehicle was then added and cells were incubated for an additional two hours. Neuronal ATP was extracted using the kit's ATP releasing agent and cellular ATP content was then determined by luminescence using a FLUOstar OPTIMA multimodal plate reader (BMG LABTECH, Inc., Cary, NC). Total ATP content was normalized to cellular protein.

### Statistics

An unpaired Student's t-test was used to compare the absolute OCRs of Type I and Type II neuronal preparations. Two-way analysis of variance (ANOVA) was employed to evaluate statistical significance among groups, with treatment and experiment number as factors. Tukey's post-hoc analysis was used to compare individual groups. P<0.05 was considered significant. Statistical analyses were carried out using SigmaPlot 12.0 (Systat Software, Inc., San Jose, CA). Results in the text are given as mean ± standard error.

## Results

**Figure 1:**
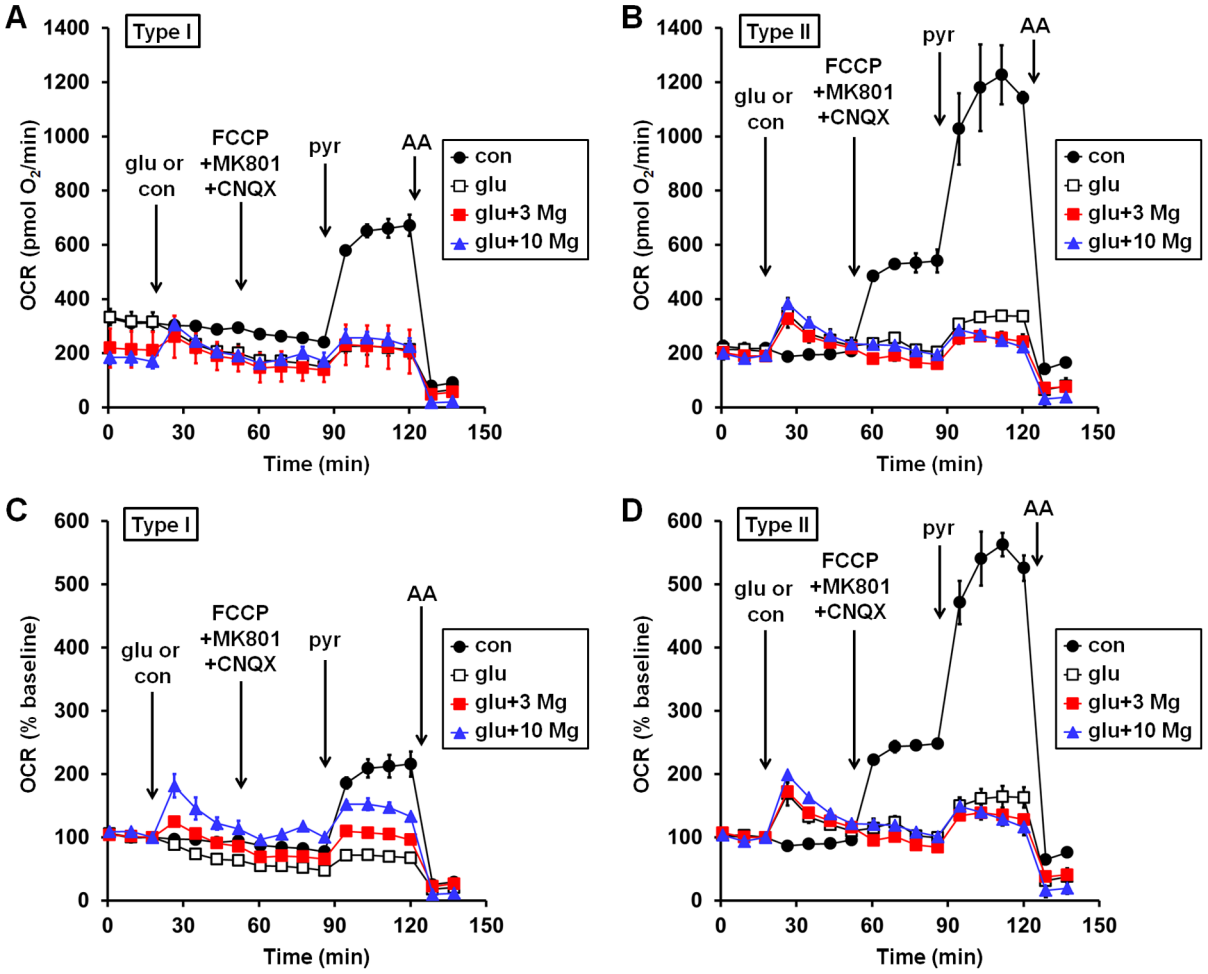
Representative Type I (A, C) and Type II (B, D) bioenergetic profiles with or without MgSO_4_ treatment. Neurons were pre-incubated for 1 hr in the absence or presence of MgSO_4_ (3 or 10 mM). Three basal O_2_ consumption rate (OCR) measurements were made in the continued absence or presence of MgSO_4_, followed by control (con) or glutamate (glu, 100 µM) addition (first arrow). NMDA receptor activation was promoted by addition of the co-agonist glycine (10 µM) with glutamate. CNQX and MK801 (10 µM each) were added to end glutamate receptor stimulation, along with FCCP (3 µM) to simultaneously uncouple mitochondria and reveal respiratory capacity in the presence of endogenous substrates (second arrow). Excess mitochondrial substrate in the form of pyruvate (pyr, 10 mM) was then supplied to reveal maximal respiratory capacity (third arrow), followed finally by the complex III inhibitor antimycin A (AA, 1 µM) to inhibit mitochondrial O_2_ consumption (fourth arrow). **(A)** and **(B)**. Absolute OCRs from Type I and Type II neurons, respectively. **(C)** and **(D)**. Baseline-normalized OCRs from Type I and Type II neurons, respectively. OCRs are normalized to the third baseline measurement prior to the addition of glutamate or control. Representative traces are mean ± SD from three wells. In some cases the error bars are smaller than the symbol size.

Oxygen consumption by 11–15 DIV rat cortical neurons was measured using the XF24 microplate-based respirometer following a 1 hr incubation in the presence or absence of MgSO_4_ (3 or 10 mM). All experiments were conducted in artificial cerebrospinal fluid (aCSF) containing a physiological concentration of Mg^2+^ (1 mM MgCl_2_). Different neuronal preparations exhibited markedly different responses to excitotoxic glutamate (100 µM, in the presence of 10 µM glycine) either in the absence or presence of MgSO_4_ pre- and co-treatment. In the absence of added MgSO_4_, populations of neurons exhibiting a decrease in O_2_ consumption rate (OCR) upon glutamate addition, or only a modest transient increase, were classified as “Type I” neurons. Examples of absolute and baseline-normalized OCR measurements from Type I cell populations (formally defined by ≤50% stimulation of OCR by glutamate) are given in Fig. 1A and C, respectively. Populations of neurons exhibiting >50% OCR stimulation in response to glutamate under the same experimental conditions were classified as Type II neurons (Fig. 1B and D). It should be noted that the Type I or Type II classification refers to the behavior of a given neuron preparation, not to the behavior of individual neurons. In addition, a preparation of neurons could only be categorized after experiments, based on the response to glutamate receptor stimulation. We failed to observe any obvious morphological differences between Type I and Type II neuronal populations.

**Figure 2:**
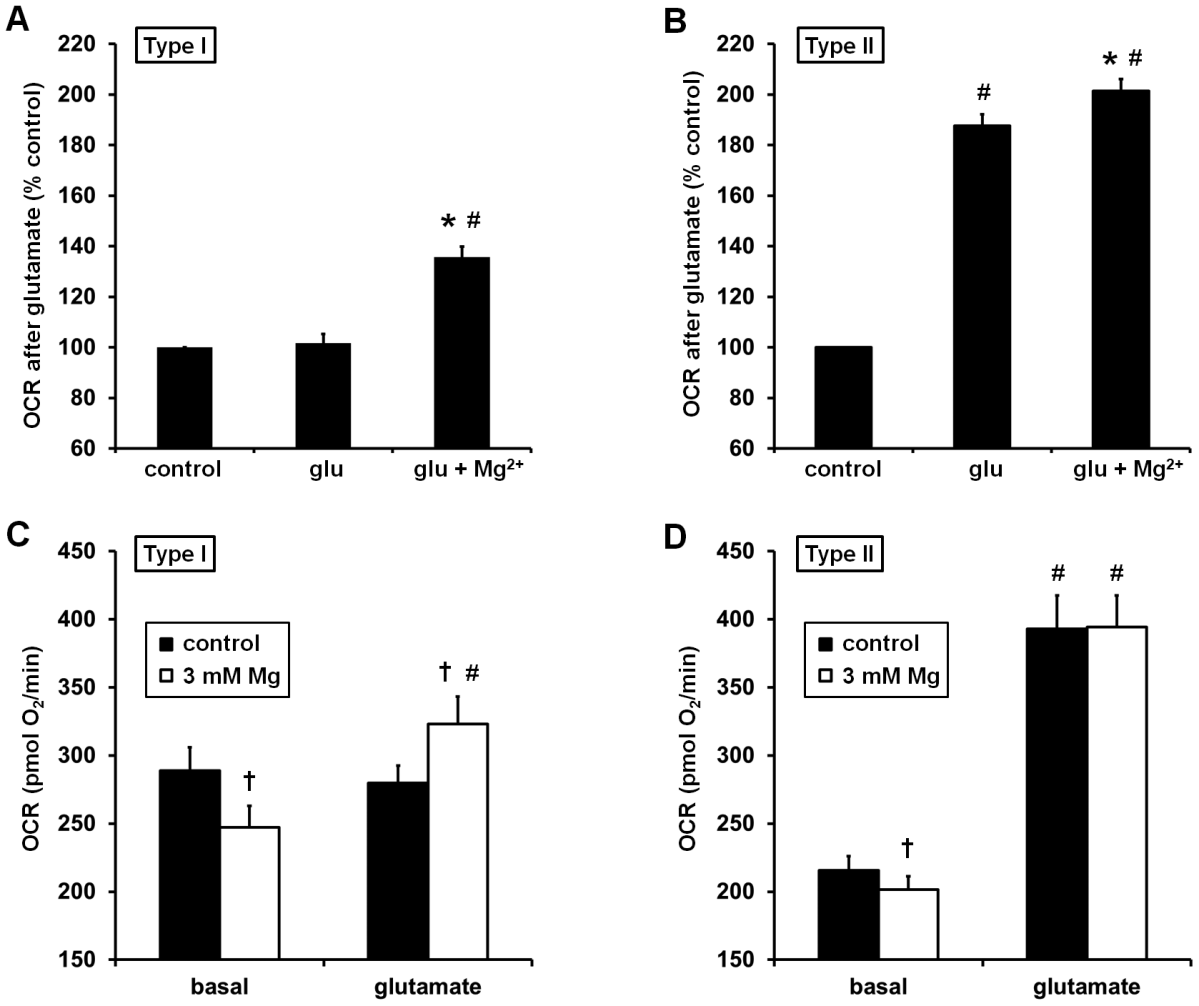
MgSO_4_ pre-treatment preferentially enhances the bioenergetic response to glutamate in Type I neurons. Normalized **(A, B)** and absolute **(C, D)** O_2_ consumption rates (OCRs) after glutamate receptor stimulation for Type I **(A, C)** and Type II **(B, D)** neuron classes are shown. OCRs in (A) and (B) are normalized to the control value corresponding to the first measurement after glutamate (glu) addition (i.e. the fourth measurement point, black circles, in the Fig. 1 traces). Results are mean ± SE from 21 (A, C) or 15 (B, D) independent experiments. *p<0.05 for glutamate plus Mg^2+^ with respect to glutamate alone (A, B). #p<0.05 for glutamate-treated with respect to control (A, B) or with respect to basal (C, D). †p<0.05 for Mg^2+^ (open bars) with respect to control (no MgSO_4_, solid bars, C, D).

Overall, glutamate did not significantly increase OCR in Type I neurons (101.6±3.7% of control, p>0.05, n = 21, Fig. 2A) whereas it increased OCR to a mean of ∼188% of the control rate in Type II neurons (Fig. 2B). However, when Type I neurons were pre-treated with MgSO_4_ (3 mM) glutamate significantly stimulated OCR to ∼136% of the control rate measured in the absence of glutamate addition (p<0.05, n = 21, Fig. 2A). MgSO_4_ also slightly but significantly enhanced the OCR stimulation of Type II neurons in response to glutamate receptor activation (from 187.6±4.6% to 201.4±4.7% of control, p<0.05, n = 15, Fig. 2B). Increasing the concentration of MgSO_4_ to 10 mM further improved stimulation of OCR by glutamate in Type I neurons (Fig. 1). Both 3 and 10 mM MgSO_4_ improved relative respiratory capacity in Type I neurons (Fig. 1C) but not in Type II neurons (Fig. 1D). Because elevating MgSO_4_ to 10 mM in humans is not therapeutically realistic, we chose to focus the remainder of our study on the effects of 3 mM MgSO_4_.

A possible reason for the failure of Type I neurons to increase OCR in response to glutamate was that energy demand was already elevated compared to Type II neurons. A higher basal demand for mitochondrial ATP should be reflected in a higher resting rate of O_2_ consumption. Substantial variability in absolute OCRs was observed due to differences in plating, viability, and cell distribution, making it difficult to accurately compare absolute basal and glutamate-stimulated rates among groups in individual experiments. However, we were able to detect statistical differences by analyzing a large number of experiments. Type I neurons had a significantly higher resting OCR (288.9±17.1 pmol O_2_/min, n = 21) compared to Type II neurons (215.7±10.5 pmol O_2_/min, n = 15), consistent with a greater energy demand at rest. MgSO_4_ significantly lowered the basal OCR of both Type I (Fig. 2C) and Type II neurons (Fig. 2D); however the magnitude of the decrease was greater for Type I neurons (14.4% compared to 6.6%). For Type I neurons, absolute OCR measured after glutamate treatment was significantly higher in the presence of MgSO_4_ (323.1±20.0 pmol O_2_/min) compared to in its absence (279.9±16.0 pmol O_2_/min). In contrast, MgSO_4_ did not significantly affect the absolute OCR after glutamate treatment in Type II neurons. OCR measured after glutamate receptor stimulation was significantly higher in Type II neurons (Fig. 2C) compared to Type I neurons (Fig. 2D) either in the absence or presence of MgSO_4_. This finding is indicative of an impaired bioenergetic response to glutamate in Type I neurons that was partially but not completely alleviated by MgSO_4_ pretreatment.

**Figure 3:**
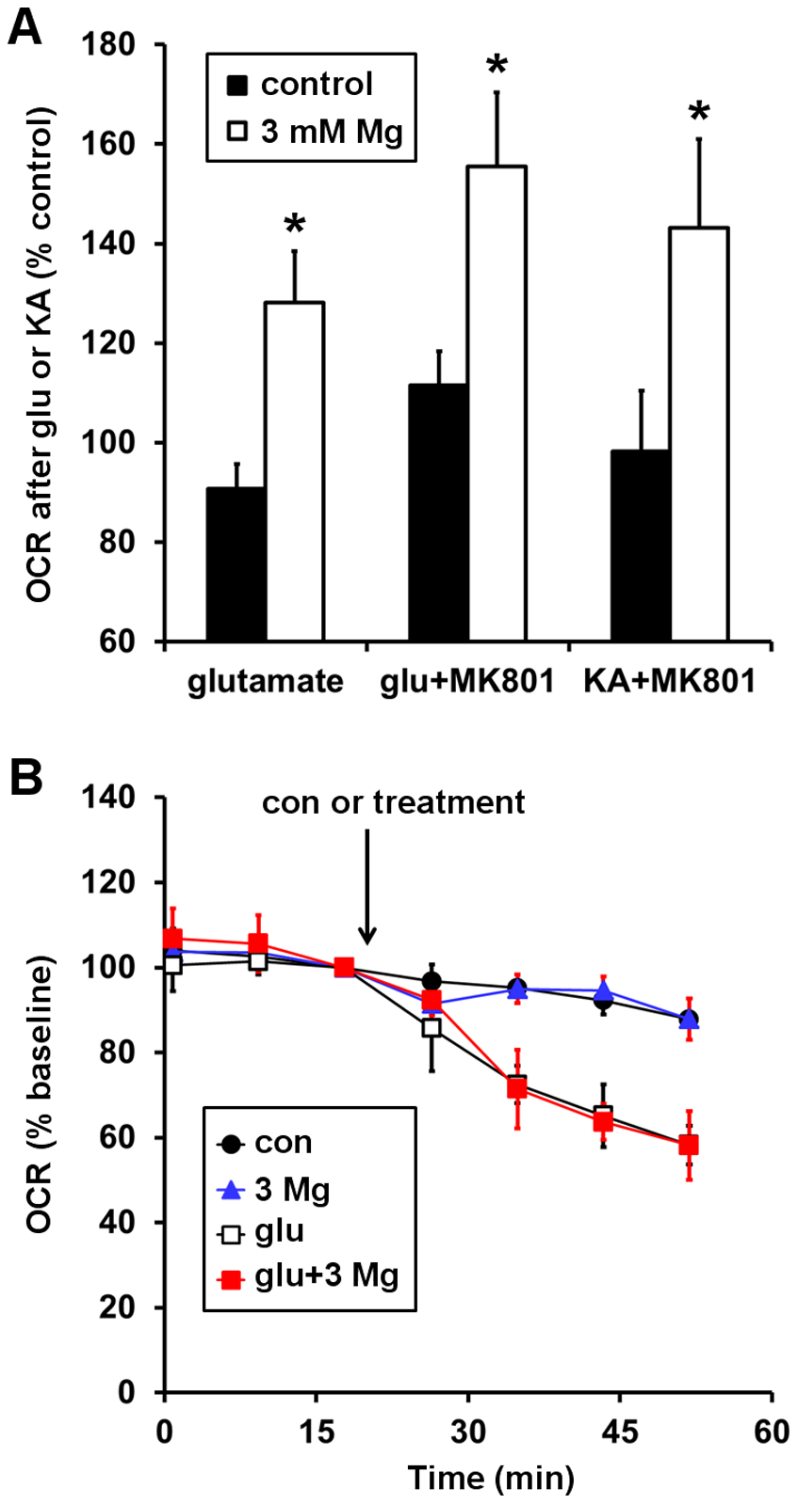
Acute Mg^2+^ action on glutamate receptors is not responsible for the enhanced glutamate bioenergetic response. The graph in **(A)** depicts normalized O_2_ consumption rates (OCRs) after exposure of Type I neurons to glutamate (glu) alone, glutamate plus MK801, or kainate (KA) plus MK801. OCRs are normalized to the control value corresponding to the first measurement after glutamate or kainate addition (i.e. the fourth measurement point in the Fig. 1 traces) *p<0.05 for Mg^2+^ (open bars) with respect to control (no MgSO_4_, solid bars). In **(B)**, basal O_2_ consumption was measured, and then control, 3 mM MgSO_4_, 100 µM glutamate, or 100 µM glutamate plus 3 mM MgSO_4_ was added at the arrow. OCRs are normalized to the third basal measurement prior to treatment. Results are mean ± SD from 3 wells.

To investigate whether an immediate action on NMDA-type glutamate receptors was required for the MgSO_4_-mediated improvement of the bioenergetic response to glutamate, we increased energy demand independent of NMDA receptor activity by selectively stimulating ionotropic AMPA-type glutamate receptors using the agonist kainate or using glutamate in the presence of the NMDA receptor antagonist MK801. MgSO_4_ pre- and co-treatment significantly improved stimulation of OCR by kainate plus MK801 or glutamate plus MK801, similar to the effect on stimulation by glutamate alone (Fig. 3A). Furthermore, under conditions permissible for NMDA receptor activation, MgSO_4_ did not improve the bioenergetic response to glutamate when added together with glutamate rather than via pre-incubation (Fig. 3B). Together, these findings demonstrate that MgSO_4_ does not influence neuronal bioenergetics via immediate, direct effects on NMDA receptors or other glutamate receptor subtypes at the time of excitotoxic (100 µM) glutamate addition.

Type I neurons typically exhibited little to no respiratory stimulation in response to the uncoupler FCCP even in the absence of glutamate treatment (Fig. 1A and C, filled circles, second arrow). This observation suggested that endogenous substrate supply was rate limiting for uncoupled respiration and might also limit respiratory stimulation in response to glutamate. Consistent with an endogenous substrate limitation, uncoupled respiration was substantially increased by addition of the cell permeable mitochondrial complex I substrate pyruvate despite the presence of abundant (15 mM) glucose (Fig. 1A and C, filled circles, third arrow). Pyruvate also increased uncoupled respiration in Type II neurons; however, type II neurons had sufficient spare respiratory capacity in the presence of endogenous substrates to respond to the increased energy demand initiated by glutamate (compare the OCR after FCCP addition under control conditions, second arrow, black circles, to the OCR after glutamate addition, first arrow, open squares).

**Figure 4:**
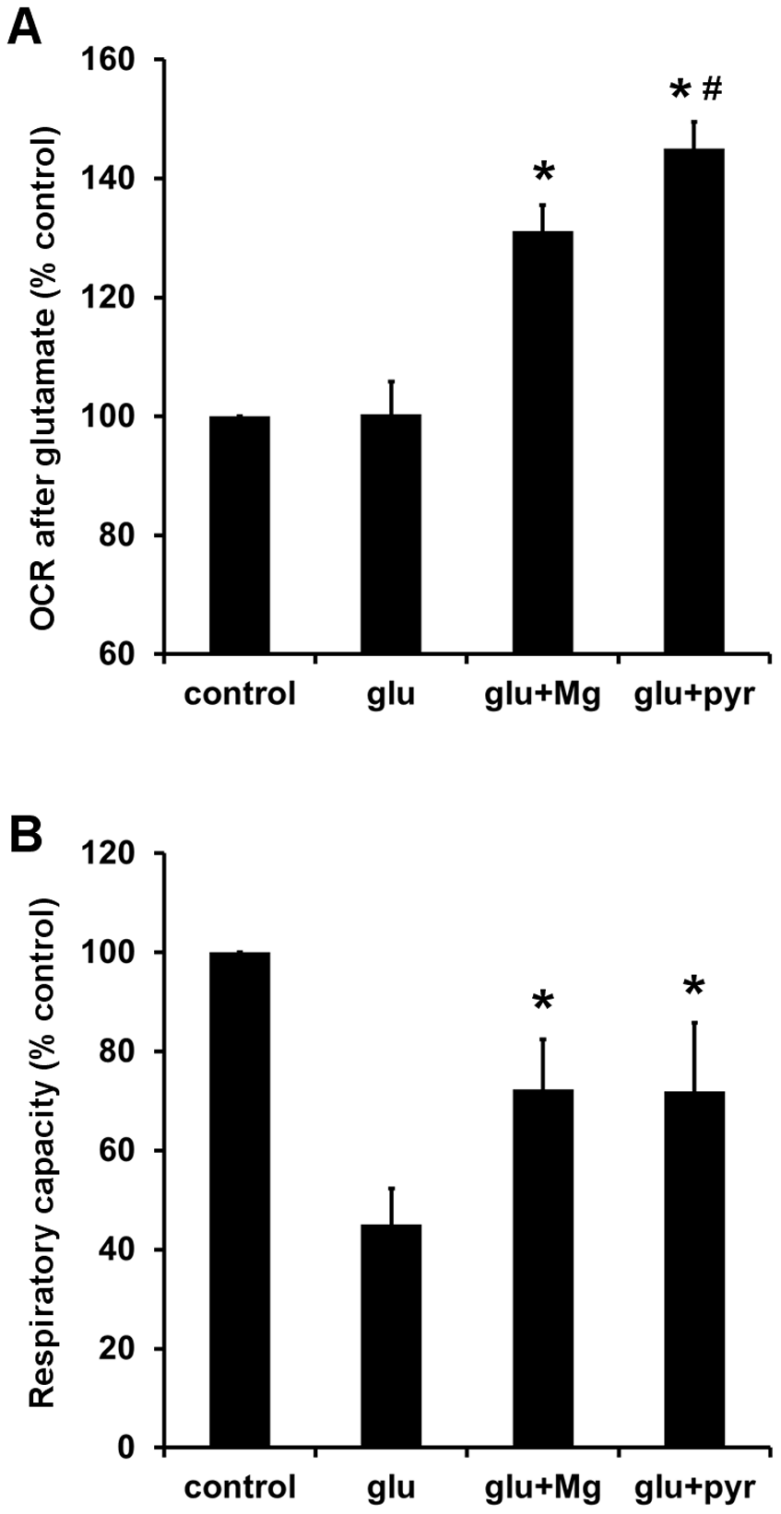
Pre-incubation with the mitochondrial substrate pyruvate mimics the beneficial bioenergetic effects of Mg^2+^ treatment. Normalized O_2_ consumption rates (OCRs) after glutamate receptor stimulation for Type I neurons are shown in **(A)**. OCRs are normalized to the control value corresponding to the first measurement after glutamate addition as in Fig. 2A and B. Results are mean ± SE from 6 independent experiments. *p<0.05 relative to control or glutamate alone. #p<0.05 for glutamate plus pyruvate relative to glutamate plus Mg^2+^. Relative respiratory capacity in Type I neurons after glutamate receptor stimulation is shown in **(B)**. OCRs are normalized to the control value corresponding to the measurement just prior to antimycin A addition in the representative time course shown in Fig. 1 (i.e. at time 120 min, subsequent to the addition of both FCCP and pyruvate). Results are mean ± SE from 5 independent experiments. *p<0.05 relative to control or glutamate alone.

To investigate the possibility that MgSO_4_ influences the bioenergetic response to glutamate by improving substrate supply, we first tested whether improving substrate supply by pyruvate pre- and co-incubation could mimic the effects of MgSO_4_ on neuronal bioenergetics. Both pyruvate (10 mM) and MgSO_4_ (3 mM) pre-treatment led to significantly higher glutamate-stimulated OCR compared to control in Type I neurons (Fig. 4A). Glutamate stimulated O_2_ consumption significantly more in the presence of pyruvate compared to in the presence of MgSO_4_ (Fig. 4A), while MgSO_4_ and pyruvate pre-treatment significantly improved the relative respiratory capacity to the same extent (Fig. 4B). Note that relative respiratory capacity was measured after the acute addition of both FCCP and pyruvate to all groups. Consequently, the protective effect of pyruvate represented an activity during the pre-incubation period and not simply the immediate effect on uncoupled respiration observed in Fig. 1.

**Figure 5:**
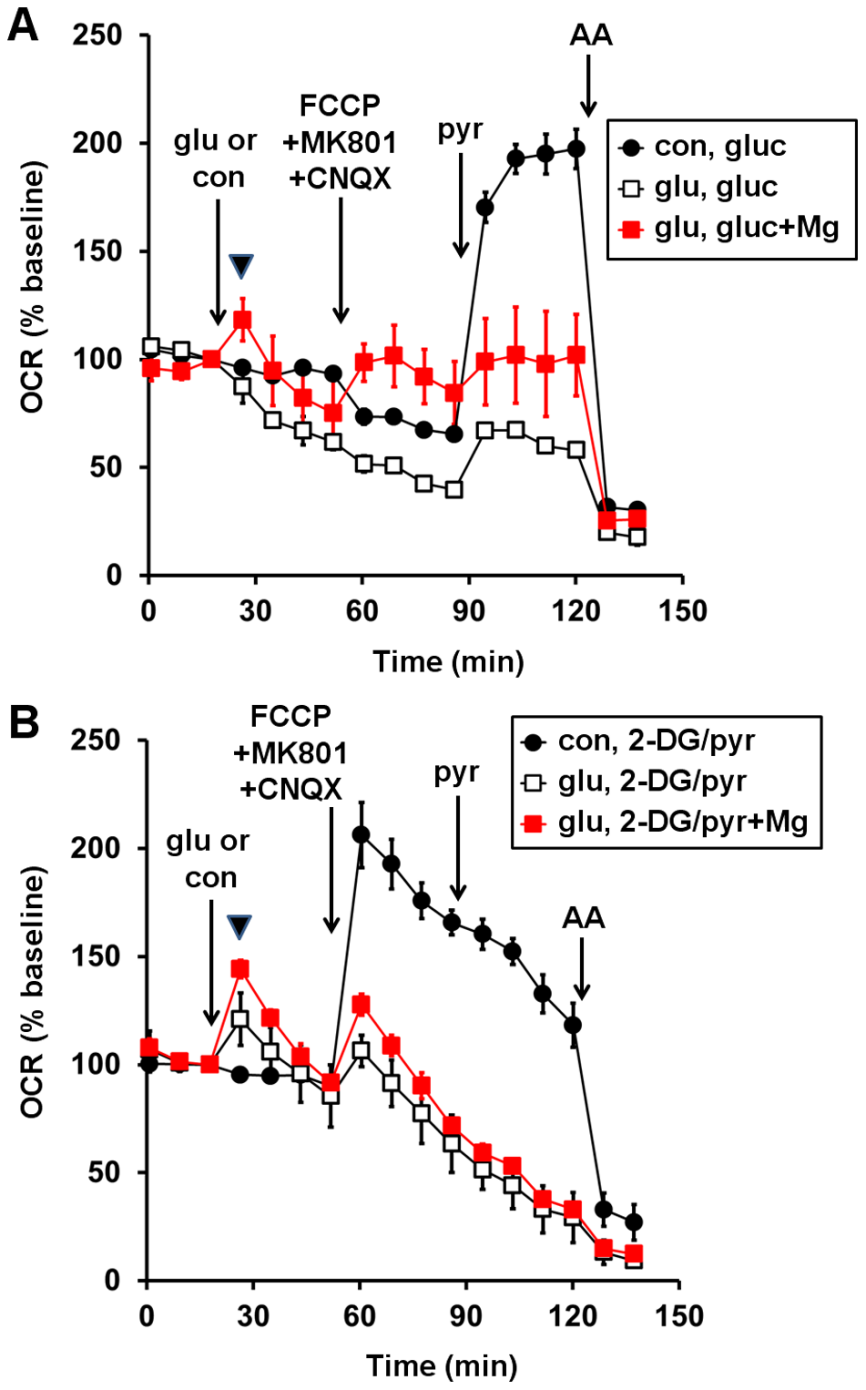
Mg^2+^ improves the bioenergetic response to glutamate independent of an effect on glycolysis. Type I neurons were pre-incubated for 1 hr in the absence or presence of MgSO_4_ (3 mM) prior to measurement of OCRs. Glutamate (glu) or control (con), FCCP+MK801+CNQX, pyruvate (pyr), and antimycin (AA) were added sequentially as in Fig. 1. Artificial CSF either contained 15 mM glucose (gluc, **A**) or 2 mM 2-deoxyglucose (2-DG) plus 10 mM pyruvate in the absence of glucose **(B)**. OCRs are normalized to the third baseline measurement prior to the addition of glutamate or control. Traces are mean ± SD from three wells and are representative of 3 independent experiments. In some cases the error bars are smaller than the symbol size.

To investigate the hypothesis that MgSO_4_ improved the bioenergetic response of Type I neurons to glutamate by stimulating glycolysis, which would in turn increase the supply of endogenous pyruvate, we asked whether MgSO_4_ would provide a bioenergetic benefit to neurons in the absence of glucose. Pyruvate (10 mM) was included in the assay medium to provide mitochondria with substrate, while 2-deoxyglucose (2-DG, 2 mM), a competitive inhibitor of glycolysis, was included to ensure that glycolysis was fully inhibited. OCR stimulation after glutamate exposure (arrowhead) was significantly higher when neurons were incubated with pyruvate and 2-DG (Fig. 5B, open squares) rather than glucose (Fig. 5A, open squares), consistent with the rate of glycolysis limiting the bioenergetic response of Type I neurons to increased energy demand (p<0.05, n = 3). Importantly, MgSO_4_ pre-treatment increased the OCR of neurons after glutamate exposure even in the absence of glycolysis (Fig. 5B), indicating that the heightened response to glutamate could not be exclusively explained by improved mitochondrial substrate delivery.

**Figure 6:**
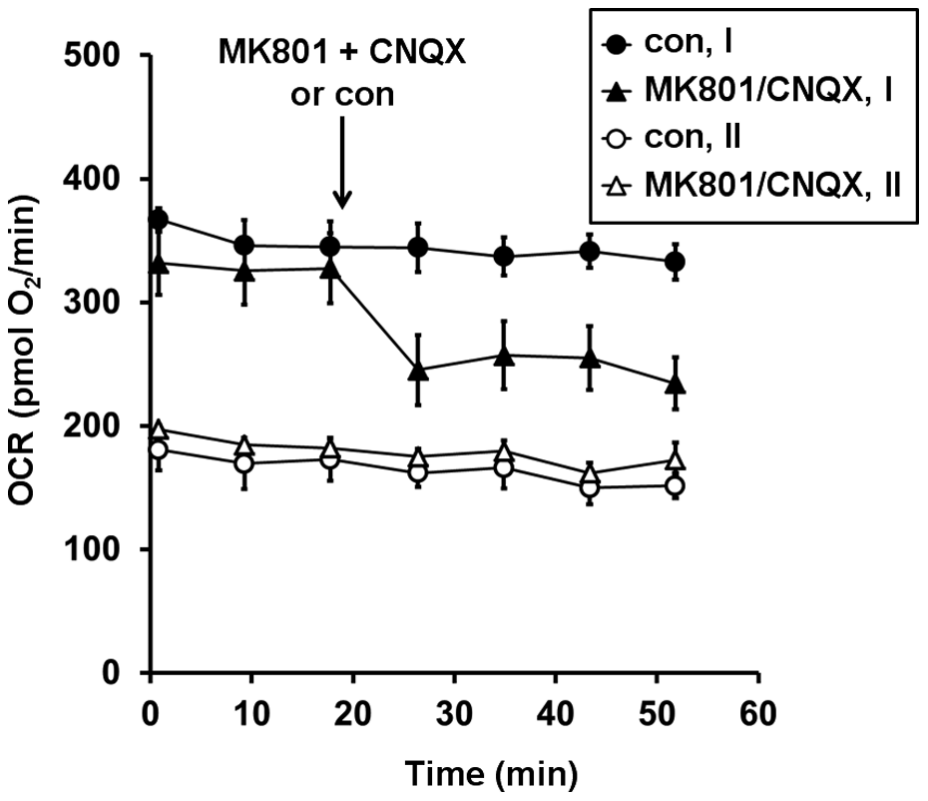
Glutamate receptor antagonists decrease basal respiration in Type I but not Type II neurons. Basal O_2_ consumption rates (OCRs) were measured, and then MK801+CNQX (10 µM each, triangles) or control (con, circles) was added at the arrow to Type I or Type II neurons (closed and open symbols, respectively). Results are mean ± SD from 3 wells.

**Figure 7:**
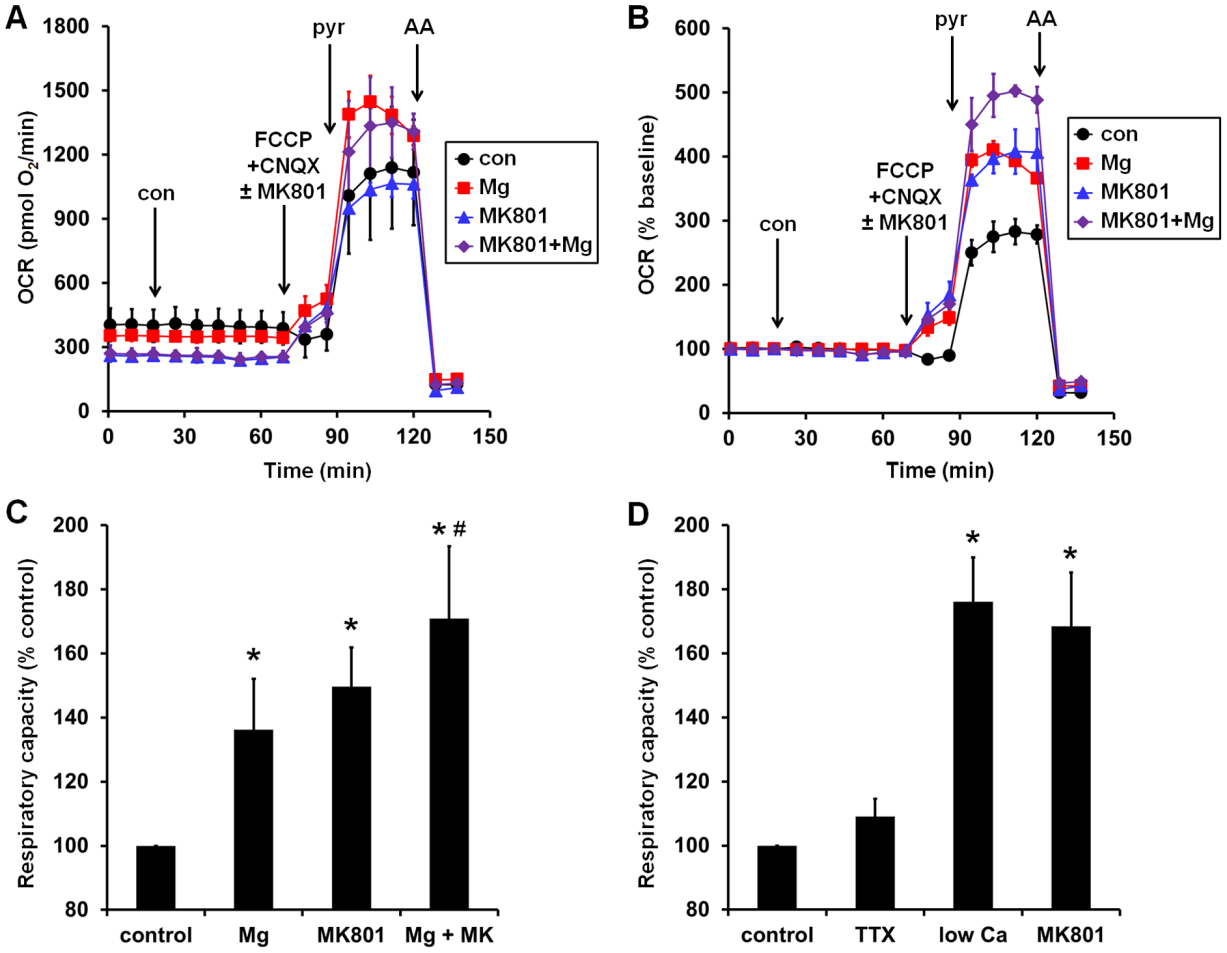
Mg^2+^ pre-treatment or preventing NMDA receptor-mediated calcium entry improves relative respiratory capacity of Type I neurons. **(A)** and **(B)** Type I neurons were pre-incubated for 1 hr in the absence or presence of MgSO_4_ (3 mM), MK801 (10 µM), or the two combined prior to measurement of OCRs. Control (con), FCCP+MK801+CNQX, pyruvate (pyr), and antimycin (AA) were added sequentially as in Fig. 1, although MK801 was omitted when already present via pre-incubation. Absolute OCRs are shown in (A) and normalized OCRs are shown in (B). OCRs in (B) are normalized to the third baseline measurement prior to the control injection. Representative traces are mean ± SD from three wells. (**(C)**) Mean relative respiratory capacities for the experiment depicted in (A) and (B). OCRs are normalized to the control value (black circles) corresponding to the measurement just prior to antimycin A addition in the representative time course shown in (B). Results are mean ± SE from 6 independent experiments. *p<0.05 relative to control. #p<0.05 relative to Mg^2+^ alone. (**(D)**) Mean relative respiratory capacities for Type I neurons pre-incubated for 1 hr in low Ca^2+^ aCSF containing 5 mM EGTA, or normal aCSF with or without the added presence of tetrodotoxin (TTX, 100 nM) or MK801 (10 µM). Treatments were also present throughout the OCR measurements. Drug additions were performed as in (A) and relative respiratory capacity was calculated as in (C). Results are mean ± SE from 3 independent experiments. *p<0.05 relative to control.

Next, we considered the possibility that although MgSO_4_ did not have an immediate effect on the bioenergetic response to glutamate (i.e., when added together with glutamate, Fig. 3B), it may modulate the response of neurons to endogenously released glutamate during the pre-incubation period. First, we tested whether application of glutamate receptor antagonists would alter basal O_2_ consumption. A combination of the NMDA and AMPA receptor antagonists MK801 and CNQX, respectively, decreased the baseline OCR in Type I but not Type II neurons (Fig. 6). This finding indicates that a major difference between Type I and Type II neurons is the occurrence of glutamate receptor stimulation under basal conditions. To test the hypothesis that the beneficial effect of MgSO_4_ treatment was due to inhibition of NMDA receptor activation during the pre-incubation period rather than at the time of excitotoxic glutamate exposure, we asked whether MgSO_4_ (3 mM), MK801 (10 µM), or the two together could increase the relative respiratory capacity of Type I neurons in the absence of exogenous glutamate treatment. Like MgSO_4_ (Fig. 2C), MK801 lowered basal OCR and enabled respiratory stimulation by uncoupler in the absence of exogenous substrate (Fig. 7A and B). In addition, both MgSO_4_ and MK801 significantly increased the relative respiratory capacity of Type I neurons measured after the addition of FCCP and pyruvate (Fig. 7C), consistent with NMDA receptor activation playing a dominant role in the impaired respiratory capacity of Type I neurons compared to Type II neurons. The respiratory capacity of Type I neurons was slightly but significantly greater when both MgSO_4_ and MK801 were added compared to MgSO_4_ treatment alone (Fig. 7C). There was also a trend toward a slight benefit of MgSO_4_ plus MK801 compared to MK801 alone that was significant if one outlying experiment was excluded (p = 0.008, n = 5). This finding suggests that although the bioenergetic protection by MgSO_4_ occurs primarily through NDMA receptor-dependent mechanisms, NMDA receptor-independent mechanisms may also make a small contribution.

A unique property of NMDA receptors compared to other glutamate receptors is their high permeability to Ca^2+^. To test whether neuronal Ca^2+^ entry was important for impairing the respiratory capacity of Type I neuronal populations, neurons were exposed to glutamate in a low Ca^2+^ aCSF (∼100 nM free Ca^2+^, see Methods) approximating cytosolic [Ca^2+^]. Low Ca^2+^ aCSF blocks Ca^2+^ entry by abolishing the Ca^2+^ gradient across the plasma membrane. Consistent with a role for NMDA-mediated Ca^2+^ entry in the respiratory impairment of Type I neurons, abolishing the Ca^2+^ gradient protected neurons to the same extent as NMDA receptor antagonism (Fig 7D). In contrast, blocking voltage-gated sodium channels with tetrodotoxin (TTX, 100 nM), which also blocks the firing of action potentials, did not significantly improve relative respiratory capacity (Fig. 7D).

**Figure 8:**
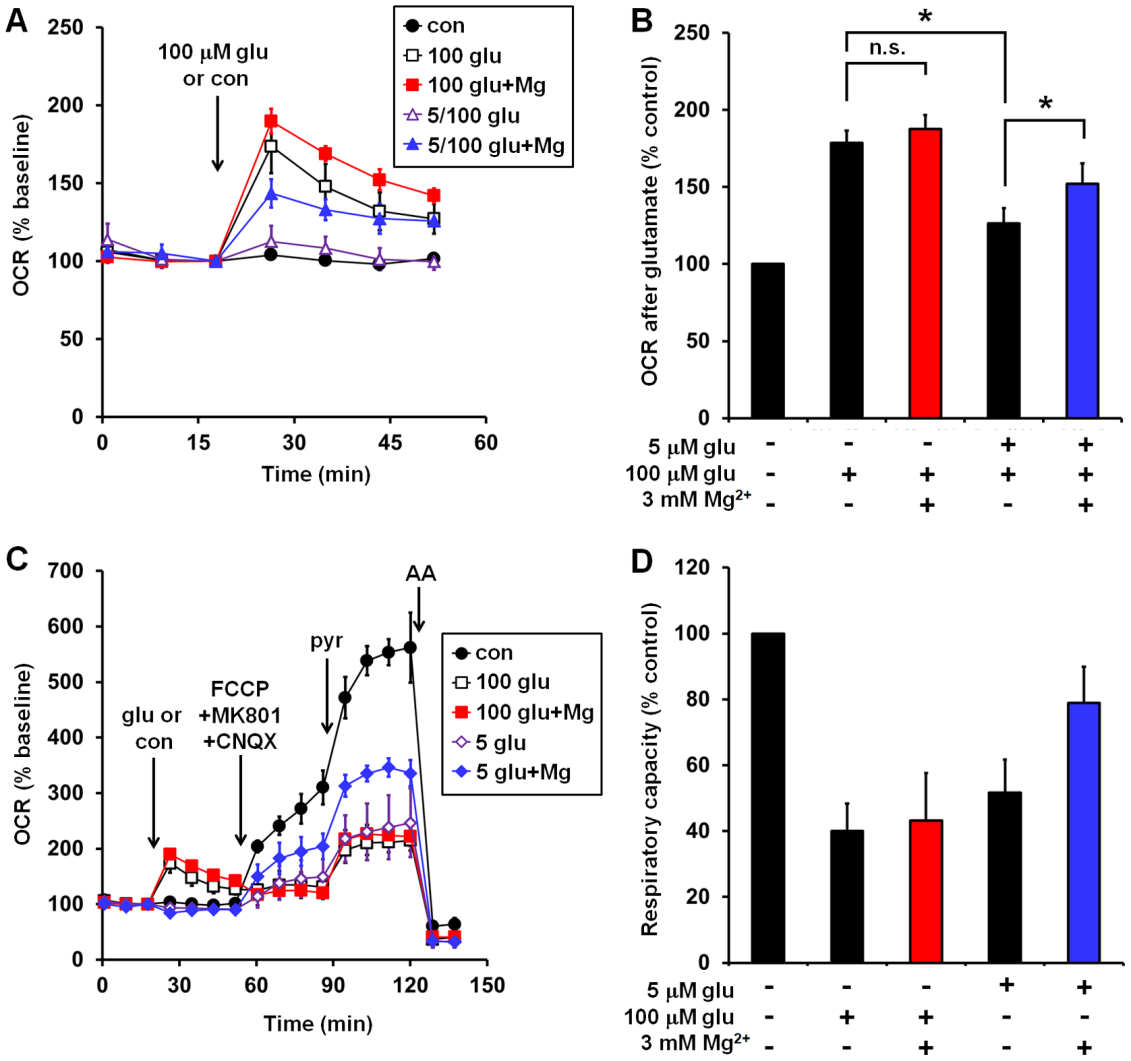
Chronic exposure of Type II neurons to 5 µM glutamate recapitulates a Type I bioenergetic phenotype. **(A)** Type II neurons were pre-incubated for 1 hr in the absence or presence of 5 µM glutamate with the added absence or presence of MgSO_4_ (3 mM) prior to measurement of OCRs. Treatments were also present throughout the OCR measurements. Glutamate (glu, 100 µM) or control (con) was added at the arrow. Numbers in the figure legend refer to the constitutively present (5 µM) or acutely added (100 µM) glutamate, respectively. OCRs are normalized to the third baseline measurement prior to the addition of glutamate or control. Representative traces are mean ± SD from three wells. **(B)** Mean normalized OCRs after glutamate receptor stimulation for the experiment depicted in (A). OCRs are normalized to the control value (black circles) corresponding to the first measurement after glutamate addition as in Fig. 2A and B. Results are mean ± SE from 3 independent experiments. *p<0.05 for the indicated comparisons. **(C)** Type II neurons were pre-incubated with or without 5 µM glutamate, 3 mM MgSO_4_, or both, as in (A), prior to measurement of OCRs. In the presence of continued treatment, three basal OCR measurements were made, followed by the subsequent sequential additions of glutamate or control, FCCP+MK801+CNQX, pyruvate (pyr), and antimycin (AA) as in Fig. 1. OCRs are normalized to the third baseline measurement prior to the addition of glutamate or control. Representative traces are mean ± SD from three wells. **(D)** Mean relative respiratory capacities for the experiment depicted in (C). OCRs are normalized to the control value (black circles) corresponding to the measurement just prior to antimycin A addition in the representative time course shown in (C). Results are mean ± SE from 3 independent experiments.

We tested whether constitutive supplementation of aCSF with a moderate (5 µM) concentration of glutamate would cause Type II neurons to adopt a Type I bioenergetic profile. Chronic glutamate receptor stimulation by 5 µM glutamate abolished stimulation of OCR by excitotoxic (100 µM) glutamate (Fig. 8A and B). However, glutamate-stimulated OCR was largely rescued by MgSO_4_ pre-treatment. In addition, MgSO_4_ improved the relative respiratory capacity of Type II neurons treated chronically with 5 µM glutamate alone by 103%, 36%, and 36% in three experiments (Fig. 8C and D, p = 0.097, n = 3) while it had no effect on the relative respiratory capacity of the same neurons treated with 100 µM glutamate acutely. Attempts to increase the sample size for this experiment yielded two Type I neuronal preparations in which MgSO_4_ improved the respiratory capacity of 5 µM glutamate-exposed neurons by 87% and 19%. Overall, MgSO_4_ significantly protected against the bioenergetic impairment caused by moderate (5 µM) chronic glutamate exposure (p<0.05, n = 5) irrespective of the initial bioenergetic phenotype (e.g. Type I vs. Type II).

**Figure 9:**
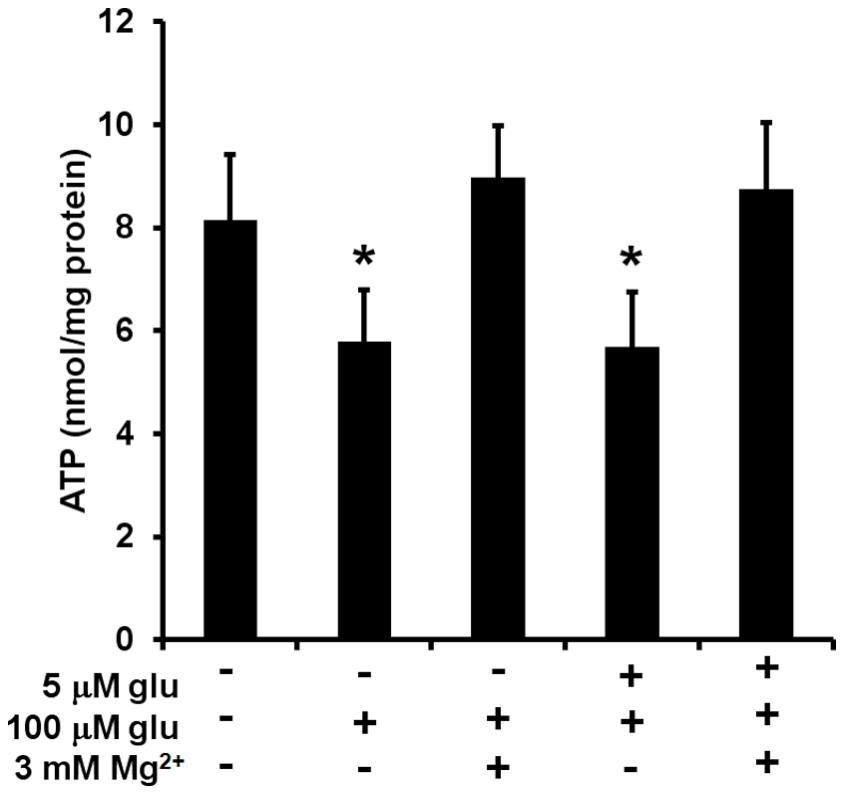
MgSO_4_ pre-treatment abolishes excitotoxic glutamate-induced ATP depletion. Neurons were pre-incubated for 1 hr in the absence or presence of 5 µM glutamate (glu) with the added absence or presence of 3 mM MgSO_4_. Neurons were then incubated for an additional 2 hr with 100 µM glu or control and total cellular ATP was measured. Results are mean ± SE from 3 independent experiments performed in triplicate and are normalized to total protein. *p<0.05 relative to control.

Finally, we tested whether MgSO_4_ could preserve ATP levels in neurons treated with excitotoxic glutamate either with or without prior 5 µM glutamate exposure. Cellular ATP was not depleted in neurons incubated with 5 µM glutamate alone, 3 mM MgSO_4_ alone, or the two combined (data not shown). However, excitotoxic (100 µM) glutamate significantly depleted neuronal ATP regardless of whether cells were previously exposed to 5 µM glutamate (Fig. 9). MgSO_4_ completely abolished the excitotoxic glutamate-induced ATP depletion both in the absence and presence of 5 µM glutamate pre-incubation (Fig. 9). Neuronal cultures in two of the three experiments were classified as Type II based on OCR measurements made prior to ATP extraction. Nevertheless, MgSO_4_ protection was observed in every experiment.

## Discussion

MgSO_4_ is already used clinically to prevent eclamptic seizures in preeclamptic women [22]. MgSO_4_ was evaluated in large clinical trials for both stroke and traumatic brain injury but without showing significant benefit, possibly due to the difficulty of overriding brain homeostatic mechanisms to achieve a neuroprotective cerebrospinal magnesium elevation [23]. Nevertheless, MgSO_4_ therapy is relatively safe and preclinical experiments point to a therapeutic benefit provided a sufficient elevation of brain magnesium can be attained [4], [5], [23]. Although the voltage-dependent magnesium blockade of calcium permeable NMDA-type glutamate receptors has been extensively investigated as a therapeutic target [16], knowledge of whether pharmacological MgSO_4_ elevation influences mitochondrial bioenergetics at the level of the intact neuron is limited.

In this study we found that preparations of primary cortical neurons could be categorized into two discrete groups based on the measured oxygen consumption rate after glutamate receptor stimulation. Type II neuronal populations exhibited a robust increase in OCR in response to glutamate (Fig. 2B) whereas Type I populations overall did not show a significant change in OCR (Fig. 2A). Glutamate receptor antagonists decreased resting OCR in Type I neurons but had no effect in Type II neurons (Fig. 6), indicating that the occurrence of ongoing endogenous glutamate receptor stimulation in Type I neurons distinguishes the two classes of neuronal preparations. Resting OCR was higher in Type I neurons (Fig. 2C) than in Type II neurons (Fig. 2D), consistent with the increased level of neuronal activity. Notably, although MgSO_4_ significantly decreased basal respiration in both Type I and Type II neurons, the magnitude of the effect was greater in Type I neurons. In addition, MgSO_4_ only improved the glutamate-stimulated respiration rate (Fig. 2C) and relative respiratory capacity (Fig. 4B) in Type I neurons. The absolute, glutamate-stimulated OCR and the uncoupled OCR were lower in Type I neurons compared to Type II neurons both with and without MgSO_4_ pre-treatment (compare Fig. 1A to Fig. 1B). This finding indicates a genuine bioenergetic impairment in Type I neurons rather than a shift in OCR exclusively due to altered energy demand. This impairment was partially but not completely ameliorated by MgSO_4_ treatment.

In Type II neurons, constitutive supplementation of aCSF with 5 µM glutamate attenuated subsequent stimulation of OCR by an excitotoxic (100 µM) concentration of glutamate (Fig. 8A and B), consistent with the possibility that the impaired bioenergetic response of Type I neurons to excitotoxic glutamate was due to pre-existing moderate glutamate receptor stimulation. As predicted, MgSO_4_ supplementation of Type II neuronal cultures exposed to chronic 5 µM glutamate restored stimulation of OCR in response to excitotoxic 100 µM glutamate and protected against a decrease in relative respiratory capacity, recapitulating a Type I phenotype.

To investigate whether the protective effects of MgSO_4_ on O_2_ consumption were associated with preservation of cellular metabolism, we studied the effects of glutamate and MgSO_4_ on neuronal ATP levels. Prolonged, three hour incubation with 5 µM glutamate did not alter the ATP profile of neurons, indicating that despite decreased respiratory capacity, the reserve capacity of these cells was still sufficient to meet the increased energy demand caused by moderate glutamate receptor activation. In contrast, excitotoxic, 100 µM glutamate significantly lowered neuronal [ATP], suggesting that the neuronal bioenergetic capacity to cope with this stress was exceeded. Remarkably, MgSO_4_ completely prevented glutamate-triggered ATP depletion (Fig. 9) even though it exhibited no protection against excitotoxic glutamate-induced attenuation of respiratory capacity in Type II neurons (Fig. 8C and D). Thus, MgSO_4_ potently preserves neuronal energy status irrespective of the Type I/II phenotype. Future measurements of basal and glutamate-stimulated intracellular calcium levels will shed further light on the mechanisms of metabolic protection by MgSO_4_.

Glutamate is the major excitatory neurotransmitter in the brain. Although several active glutamate uptake mechanisms exist, extracellular glutamate is always present in the brain to some degree. Withdrawal of extracellular magnesium from cultures of primary neurons causes epileptiform bursts of neuronal activity, intracellular calcium oscillations, mitochondrial depolarization, and a slow, NMDA receptor-dependent cell death [24], [25]. These findings indicate that a critical level of extracellular Mg^2+^ is essential to prevent seizure-like activity. Hypomagnesemia, as measured by serum Mg^2+^ concentration, is correlated to the severity of neurological deficits in human patients following stroke [26], traumatic brain injury [27], and subarachnoid hemorrhage [28]. Thus, therapeutic MgSO_4_ may be beneficial by repleting normal extracellular Mg^2+^ and/or by achieving an elevated, neuroprotective Mg^2+^ concentration beyond the normal level.

Type I neurons behaved as if the 1 mM MgCl_2_ present in aCSF was insufficient to suppress endogenous NMDA receptor activity. In fact, in the absence of added glutamate, supplementation of aCSF with 3 mM MgSO_4_ increased the relative respiratory capacity of Type I neurons to the same degree as the NMDA receptor antagonist MK801 (Fig. 7B and C, measured after a 2.5 hr incubation in aCSF). This finding demonstrates that NMDA receptor activity indeed influences mitochondrial bioenergetics in Type I neurons at rest. The effect of endogenous NMDA receptor activity was calcium-mediated, as demonstrated by the removal of extracellular calcium, but not due to action potential-dependent glutamate release, as shown by the inability of tetrodotoxin to increase relative respiratory capacity (Fig. 7D).

Although MgSO_4_ pretreatment was effective, MgSO_4_ co-treatment failed to protect Type I neurons from excitotoxic glutamate-induced bioenergetic decline (Fig. 3B). The ability of Mg^2+^ to block NMDA receptors is voltage-dependent [15]. Excitotoxic concentrations of glutamate strongly depolarize neurons via AMPA receptor activation, rendering Mg^2+^ incapable of impeding NMDA receptor channel activity. However, it is possible that the more modest levels of glutamate receptor activation due to chronic, endogenous activity in Type I neurons do not depolarize cells sufficiently to prevent voltage-dependent Mg^2+^ inhibition.

A major limitation of our study is that to date, we have been unable to determine the source(s) of variability in our neuronal preparations that cause a Type I or Type II phenotype with more or less endogenous glutamate receptor activity. Because tetrodotoxin did not mimic the protective effect of MK801, the origin of endogenous extracellular glutamate in Type I neuronal preparations was not action potential-dependent vesicular release. MK801 or enzymatic glutamate removal but not tetrodotoxin protected primary rat cortical neurons from death induced by the glutamate transport inhibitor L-*trans*-pyrrolidine-2,4-dicarboxylate [29]. Extracellular glutamate was measured at 3 µM in that study, similar to the concentration added to Type II neurons to recapitulate the Type I phenotype (5 µM, Fig. 8). Thus, it is possible that the elevated endogenous glutamate receptor activity in Type I preparations was due to the presence of neurons with impaired glutamate transport. Heterogeneity in the properties of cultured neurons has been noted previously. Variability in commercial B27 (Invitrogen), a supplement used to maintain neuronal cultures in place of serum, was shown to influence the number of neuronal filopodia, dendritic spines, and degenerating axons over extended culture [30]. In addition, it is well-established that the sensitivity of neurons to glutamate is stochastic, with individual neurons even on a single coverslip displaying dramatically different times to calcium deregulation in response to excitotoxic glutamate treatment [11], [31]. Neuronal cultures with properties intermediate to Type I and Type II would probably be the most physiologically relevant, with a basal tone of glutamate receptor stimulation that utilizes greater mitochondrial respiratory capacity than in Type II neurons but to an extent that does not elicit the NMDA receptor-dependent bioenergetic impairment of Type I neurons. Further work is clearly needed to elucidate the source of the excess basal glutamate receptor activity in Type I neurons and optimize cultures for consistency. Nevertheless, Type I cultures provided a valuable model to study the bioenergetic consequences of an ongoing moderate level of glutamate receptor stimulation that we were able to mimic in Type II cultures by constitutive glutamate (5 µM) supplementation.

Pyruvate protected rat cortical neurons from death induced by low extracellular Mg^2+^ [25]. Like MgSO_4_, pyruvate (10 mM) ameliorated the bioenergetic consequences of chronic glutamate exposure in our study (Fig. 4). However, the protective effect of MgSO_4_ could not be explained simply by increased provision of pyruvate to mitochondria through glycolysis because a beneficial effect of extracellular Mg^2+^ was observed even when abundant exogenous pyruvate was supplied in the absence of glycolysis (Fig. 5B). Nevertheless, the protective effect of pyruvate when glucose was present (Fig. 4) suggests that the inability of endogenous mitochondrial substrate supply to meet elevated energy demand contributes to the deleterious consequences of chronic glutamate receptor activity. Further studies are needed to delineate the extent to which effects of MgSO_4_ on NMDA receptor activity vs. other effects on cellular metabolism contribute to its protective actions.

Overall, our studies show that a clinically pharmacologic MgSO_4_ concentration protects against a loss of neuronal mitochondrial spare respiratory capacity caused by chronic, endogenous glutamate receptor stimulation. In addition, MgSO_4_ improves the ability of mitochondria to respond to an increase in energy demand elicited by a subsequent excitotoxic concentration of glutamate. Optimization of a protocol for delivering MgSO_4_ across the blood-brain-barrier holds promise for the protection of neurons from stroke, cardiac arrest, traumatic brain injury, or subarachnoid hemorrhage-induced metabolic failure.

## References

[1] Panov A, Scarpa A (1996) Independent modulation of the activity of alpha-ketoglutarate dehydrogenase complex by Ca2+ and Mg2+. Biochemistry 35: 427–432. 8555212 10.1021/bi952101t

[2] Rodriguez-Zavala JS, Moreno-Sanchez R (1998) Modulation of oxidative phosphorylation by Mg2+ in rat heart mitochondria. J Biol Chem 273: 7850–7855. 9525878 10.1074/jbc.273.14.7850

[3] Xu M, Dai W, Deng X (2002) Effects of magnesium sulfate on brain mitochondrial respiratory function in rats after experimental traumatic brain injury. Chin J Traumatol 5: 361–364. 12443578

[4] Heath DL, Vink R (1999) Optimization of magnesium therapy after severe diffuse axonal brain injury in rats. J Pharmacol Exp Ther 288: 1311–1316. 10027872

[5] Zhu H, Meloni BP, Bojarski C, Knuckey MW, Knuckey NW (2005) Post-ischemic modest hypothermia (35 degrees C) combined with intravenous magnesium is more effective at reducing CA1 neuronal death than either treatment used alone following global cerebral ischemia in rats. Exp Neurol 193: 361–368. 15869938 10.1016/j.expneurol.2005.01.022

[6] Lampl Y, Geva D, Gilad R, Eshel Y, Ronen L, et al (1998) Cerebrospinal fluid magnesium level as a prognostic factor in ischaemic stroke. J Neurol 245: 584–588. 9758295 10.1007/s004150050249

[7] Arundine M, Tymianski M (2004) Molecular mechanisms of glutamate-dependent neurodegeneration in ischemia and traumatic brain injury. Cell Mol Life Sci 61: 657–668. 15052409 10.1007/s00018-003-3319-x PMC11138528

[8] Jekabsons MB, Nicholls DG (2004) In situ respiration and bioenergetic status of mitochondria in primary cerebellar granule neuronal cultures exposed continuously to glutamate. J Biol Chem 279: 32989–33000. 15166243 10.1074/jbc.M401540200

[9] Yadava N, Nicholls DG (2007) Spare respiratory capacity rather than oxidative stress regulates glutamate excitotoxicity after partial respiratory inhibition of mitochondrial complex I with rotenone. J Neurosci 27: 7310–7317. 17611283 10.1523/JNEUROSCI.0212-07.2007 PMC6794596

[10] Gleichmann M, Collis LP, Smith PJ, Mattson MP (2009) Simultaneous single neuron recording of O2 consumption, [Ca2+]i and mitochondrial membrane potential in glutamate toxicity. J Neurochem 109: 644–655. 19226367 10.1111/j.1471-4159.2009.05997.x PMC2805059

[11] Nicholls DG (2009) Spare respiratory capacity, oxidative stress and excitotoxicity. Biochem Soc Trans 37: 1385–1388. 19909281 10.1042/BST0371385

[12] Castilho RF, Hansson O, Ward MW, Budd SL, Nicholls DG (1998) Mitochondrial control of acute glutamate excitotoxicity in cultured cerebellar granule cells. J Neurosci 18: 10277–10286. 9852565 10.1523/JNEUROSCI.18-24-10277.1998 PMC6793348

[13] Stout AK, Raphael HM, Kanterewicz BI, Klann E, Reynolds IJ (1998) Glutamate-induced neuron death requires mitochondrial calcium uptake. Nat Neurosci 1: 366–373. 10196525 10.1038/1577

[14] Abramov AY, Duchen MR (2008) Mechanisms underlying the loss of mitochondrial membrane potential in glutamate excitotoxicity. Biochim Biophys Acta 1777: 953–964. 18471431 10.1016/j.bbabio.2008.04.017

[15] Nowak L, Bregestovski P, Ascher P, Herbet A, Prochiantz A (1984) Magnesium gates glutamate-activated channels in mouse central neurones. Nature 307: 462–465. 6320006 10.1038/307462a0

[16] Hallak M (1998) Effect of parenteral magnesium sulfate administration on excitatory amino acid receptors in the rat brain. Magnes Res 11: 117–131. 9675756

[17] Stoica BA, Movsesyan VA, Knoblach SM, Faden AI (2005) Ceramide induces neuronal apoptosis through mitogen-activated protein kinases and causes release of multiple mitochondrial proteins. Mol Cell Neurosci 29: 355–371. 15905098 10.1016/j.mcn.2005.02.009

[18] Yakovlev AG, Ota K, Wang G, Movsesyan V, Bao WL, et al (2001) Differential expression of apoptotic protease-activating factor-1 and caspase-3 genes and susceptibility to apoptosis during brain development and after traumatic brain injury. J Neurosci 21: 7439–7446. 11567033 10.1523/JNEUROSCI.21-19-07439.2001 PMC6762901

[19] Clerc P, Polster BM (2012) Investigation of mitochondrial dysfunction by sequential microplate-based respiration measurements from intact and permeabilized neurons. PLoS ONE 7: e34465. 22496810 10.1371/journal.pone.0034465 PMC3319583

[20] Almeida A, Bolanos JP (2001) A transient inhibition of mitochondrial ATP synthesis by nitric oxide synthase activation triggered apoptosis in primary cortical neurons. J Neurochem 77: 676–690. 11299330 10.1046/j.1471-4159.2001.00276.x

[21] Wu M, Neilson A, Swift AL, Moran R, Tamagnine J, et al (2007) Multiparameter metabolic analysis reveals a close link between attenuated mitochondrial bioenergetic function and enhanced glycolysis dependency in human tumor cells. Am J Physiol Cell Physiol 292: C125–C136. 16971499 10.1152/ajpcell.00247.2006

[22] Greene MF (2003) Magnesium sulfate for preeclampsia. N Engl J Med 348: 275–276. 12540639 10.1056/NEJMp020166

[23] McKee JA, Brewer RP, Macy GE, Borel CO, Reynolds JD, et al (2005) Magnesium neuroprotection is limited in humans with acute brain injury. Neurocrit Care 2: 342–351. 16159086 10.1385/NCC:2:3:342

[24] Deshpande LS, Lou JK, Mian A, Blair RE, Sombati S, et al (2008) Time course and mechanism of hippocampal neuronal death in an in vitro model of status epilepticus: role of NMDA receptor activation and NMDA dependent calcium entry. Eur J Pharmacol 583: 73–83. 18289526 10.1016/j.ejphar.2008.01.025 PMC2323609

[25] Kovac S, Domijan AM, Walker MC, Abramov AY (2012) Prolonged seizure activity impairs mitochondrial bioenergetics and induces cell death. J Cell Sci 125: 1796–1806. 22328526 10.1242/jcs.099176 PMC4195235

[26] Altura BT, Memon ZI, Zhang A, Cheng TP, Silverman R, et al (1997) Low levels of serum ionized magnesium are found in patients early after stroke which result in rapid elevation in cytosolic free calcium and spasm in cerebral vascular muscle cells. Neurosci Lett 230: 37–40. 9259458 10.1016/s0304-3940(97)00471-0

[27] Memon ZI, Altura BT, Benjamin JL, Cracco RQ, Altura BM (1995) Predictive value of serum ionized but not total magnesium levels in head injuries. Scand J Clin Lab Invest 55: 671–677. 8903837 10.3109/00365519509075397

[28] van den Bergh WM, Algra A, van der Sprenkel JW, Tulleken CA, Rinkel GJ (2003) Hypomagnesemia after aneurysmal subarachnoid hemorrhage. Neurosurgery 52: 276–281. 12535355 10.1227/01.neu.0000043984.42487.0e

[29] Blitzblau R, Gupta S, Djali S, Robinson MB, Rosenberg PA (1996) The glutamate transport inhibitor L-trans-pyrrolidine-2,4-dicarboxylate indirectly evokes NMDA receptor mediated neurotoxicity in rat cortical cultures. Eur J Neurosci 8: 1840–1852. 8921275 10.1111/j.1460-9568.1996.tb01328.x

[30] Chen Y, Stevens B, Chang J, Milbrandt J, Barres BA, et al (2008) NS21: re-defined and modified supplement B27 for neuronal cultures. J Neurosci Methods 171: 239–247. 18471889 10.1016/j.jneumeth.2008.03.013 PMC2678682

[31] Gerencser AA, Mark KA, Hubbard AE, Divakaruni AS, Mehrabian Z, et al (2009) Real-time visualization of cytoplasmic calpain activation and calcium deregulation in acute glutamate excitotoxicity. J Neurochem 110: 990–1004. 19493161 10.1111/j.1471-4159.2009.06194.x PMC2745075

